# Supplementary figures and images for: TLR4 competence and mouse models of leptospirosis
Source: bioRxiv. 2025 Feb 4:2025.02.03.636333. Preprint. [Version 2] doi: 10.1101/2025.02.03.636333 (PMC11838568; doi:10.1101/2025.02.03.636333)

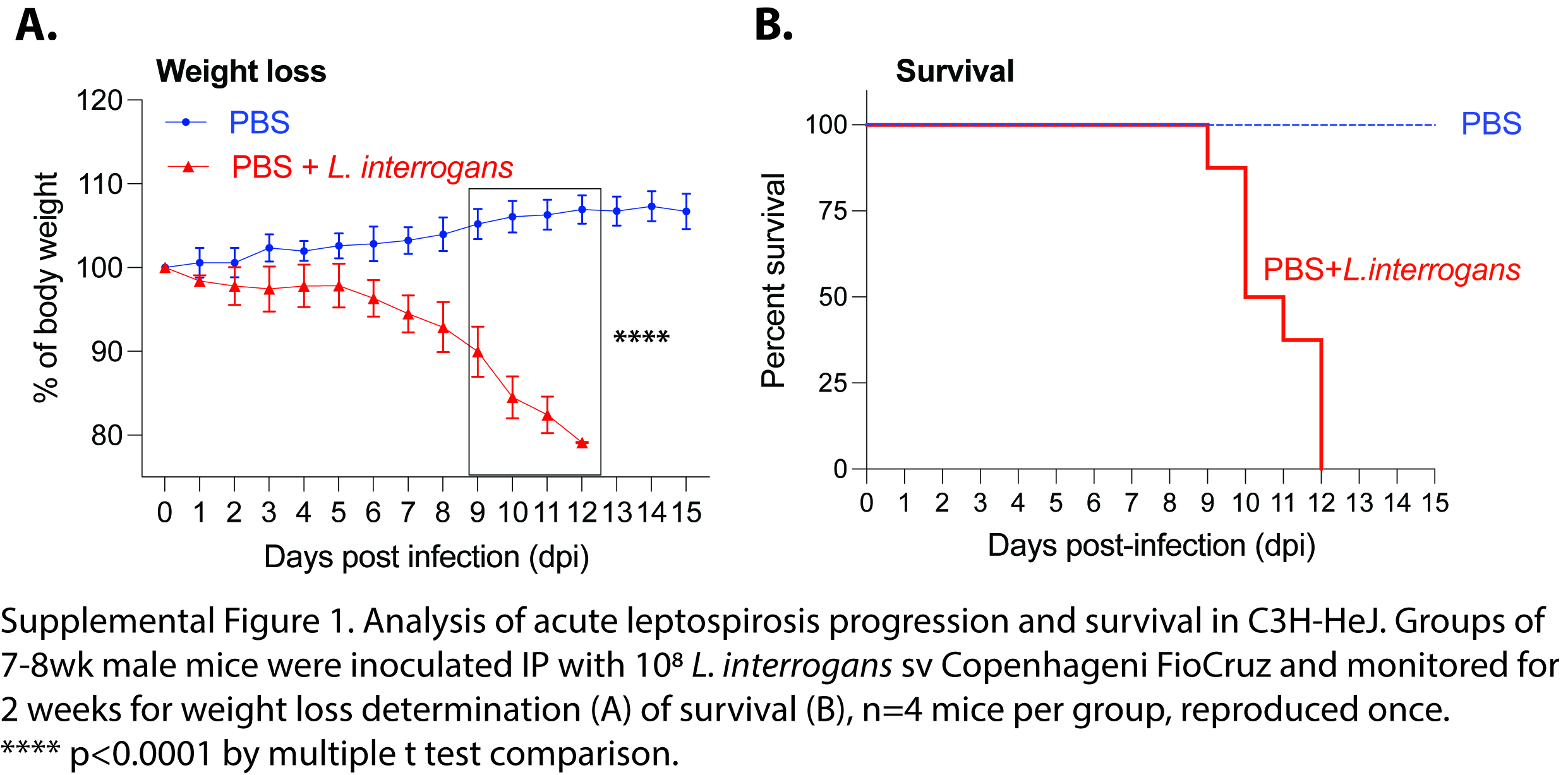

Supplement: Supplement 1 [file media-1.tif]
